# Supplementary material for: Human macrophages survive and adopt activated genotypes in living zebrafish
Source: Sci Rep. 2019 Feb 11;9:1759. doi: 10.1038/s41598-018-38186-y (PMC6370805; doi:10.1038/s41598-018-38186-y)
Supplement: Supplementary file 1 — Supplementary Info [file 41598_2018_38186_MOESM1_ESM.docx]

**Human macrophages survive and adopt activated genotypes in living zebrafish**

Colin D Paul^1#^, Alexus Devine^1#^, Kevin Bishop^2^, Qing Xu^3^, William J Wulftange^4^, Hannah Burr^1^, Kathryn M Daly^1^, Chaunte Lewis^1^, Daniel S Green^5^, Jack R Staunton^1^, Swati Choksi^3^, Zheng-Gang Liu^3^, Raman Sood^2^, and Kandice Tanner^1*^

^1^ Laboratory of Cell Biology, Center for Cancer Research, National Cancer Institute, National Institutes of Health

^2^ Zebrafish Core, National Human Genomic Research Institute, National Institutes of Health

^3^Laboratory of Immune Cell Biology, Center for Cancer Research, National Cancer Institute, National Institutes of Health

^4^National Institute of Biomedical Imaging and Bioengineering, National Institutes of Health

^5^Women’s Malignancy Branch, Center for Cancer Research, National Cancer Institute, National Institutes of Health

^#^ co-first authors

^*^ To whom correspondence should be addressed: Kandice Tanner, Ph.D., 37 Convent Dr., Bethesda, MD 20852. Email: [kandice.tanner@nih.gov](mailto:kandice.tanner@nih.gov)

**
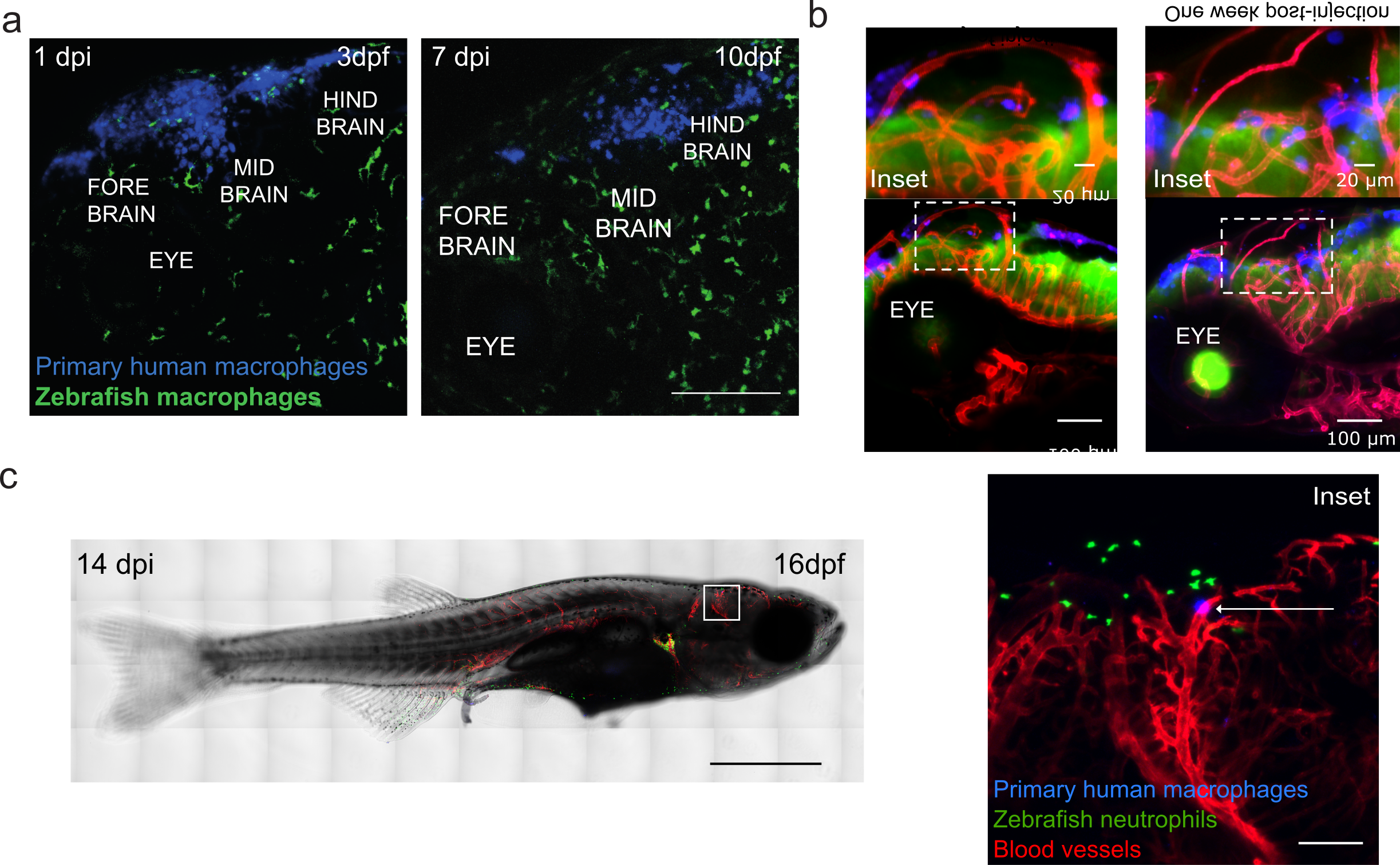
**

**Supplemental Figure 1- Human immortalized monocytes and primary macrophages survive *in vivo* for up to two weeks.** (a) Micrographs of 3D projections showing distribution and survival of human primary macrophages (blue) injected into the hind brain of transgenic zebrafish larvae, mpeg:GFP (zebrafish macrophages-green), at 2 days post fertilization (2 dpf). Left panel shows 1 day post injection (1 dpi) when larvae is 3 dpf and right panel, same fish 7 dpi when at 9 dpf. Scale bar = 100 µm. (b) U937 human monocytes (blue) were injected into the hind brain of transgenic zebrafish larvae, GFAP:GFP (astrocytes-green)/flk:mCherry (vessels-red) at 2 days post fertilization (2 dpf). Left panel shows 3D projection of injected cells on the day of injection, with detail shown in the inset. Right panel illustrates that these cells can survive over time, showing an injected larva one week after injection at 9 dpf. (c) Micrographs show that cells can persist for up to 2 weeks after injection into mpx:GFP (neutrophils-green)/flk:mCherry (vessels-red) at 2 days post fertilization (2 dpf). Left panel micrograph shows tiled image of the 16 dpf zebrafish, and white square highlights region of interest in the zebrafish brain. Scale bar = 500 µm. Right panel shows the indicated inset, where the white arrow indicates the human cell in contact with the blood vessel in the mid-hind brain. Scale bar = 100 µm.


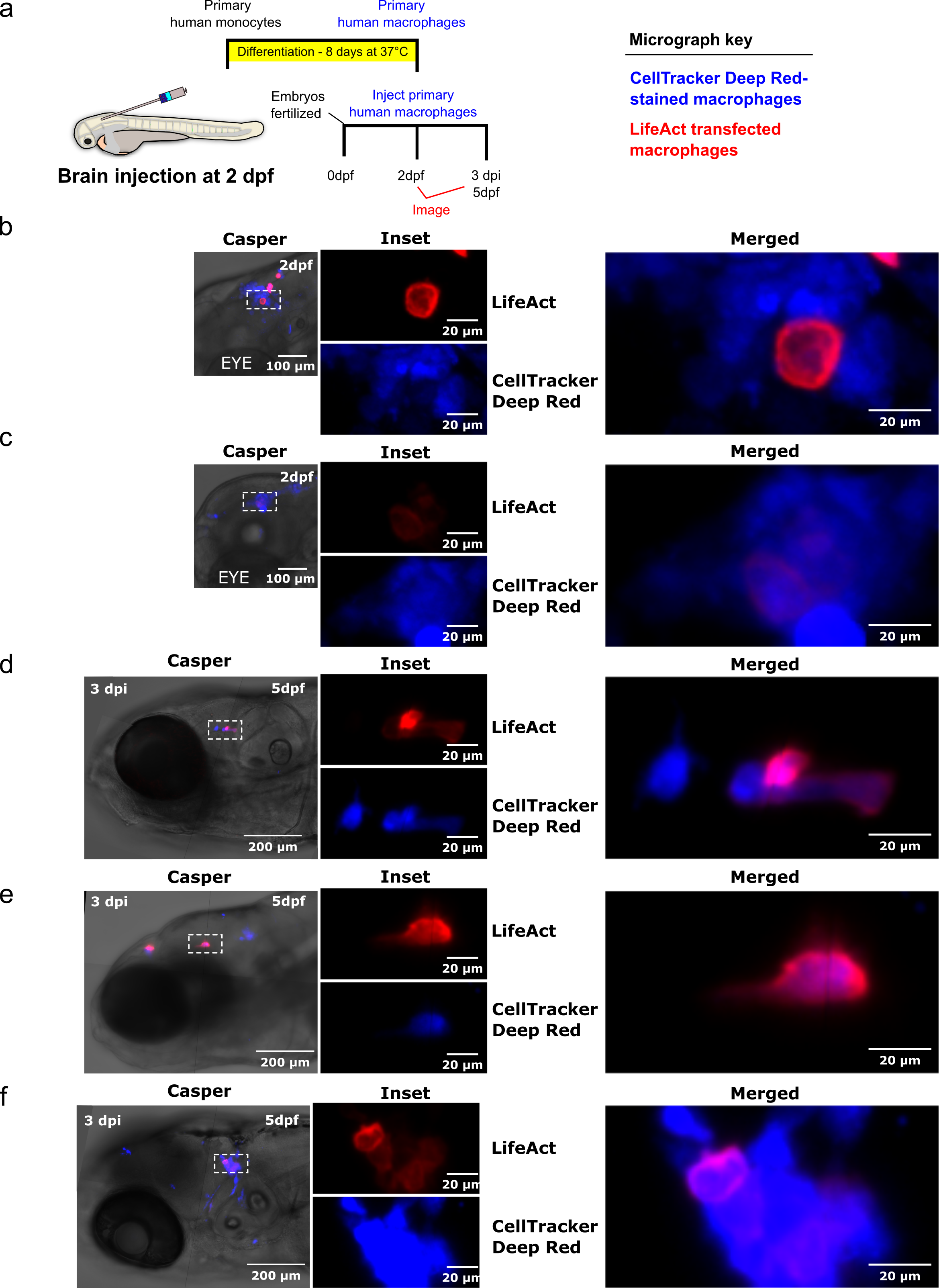


**Supplemental Figure 2- Injected immune cells transduced with LifeAct are also stained with membrane dye.** (a) Schematic of experimental design: primary monocytes were differentiated into macrophages at 37°C and transduced with LifeAct adenovirus (LifeAct positive cells displayed in red) one day prior to injection. Macrophages were co-stained with CellTracker Deep Red (displayed in blue) before injection into the brain of Casper zebrafish at 2 days post fertilization (dpf) and imaged ~4 h following injection and at 3 days post injection (dpi). (b,c) Micrographs of 3D projections showing co-staining with CellTracker Deep Red for all cells expressing LifeAct on the day of injection (2 dpf). Only a subpopulation of cells was successfully transduced. Inset locations and scales are indicated. Merged images of individual channels are also shown. (d-f) Micrographs of 3D projections showing co-staining with CellTracker Deep Red for all cells expressing LifeAct at 3 dpi. Only a subpopulation of cells was successfully transduced. All cells expressing LifeAct were also stained with CellTracker. Inset locations and scales are indicated. Merged images of individual channels are also shown.


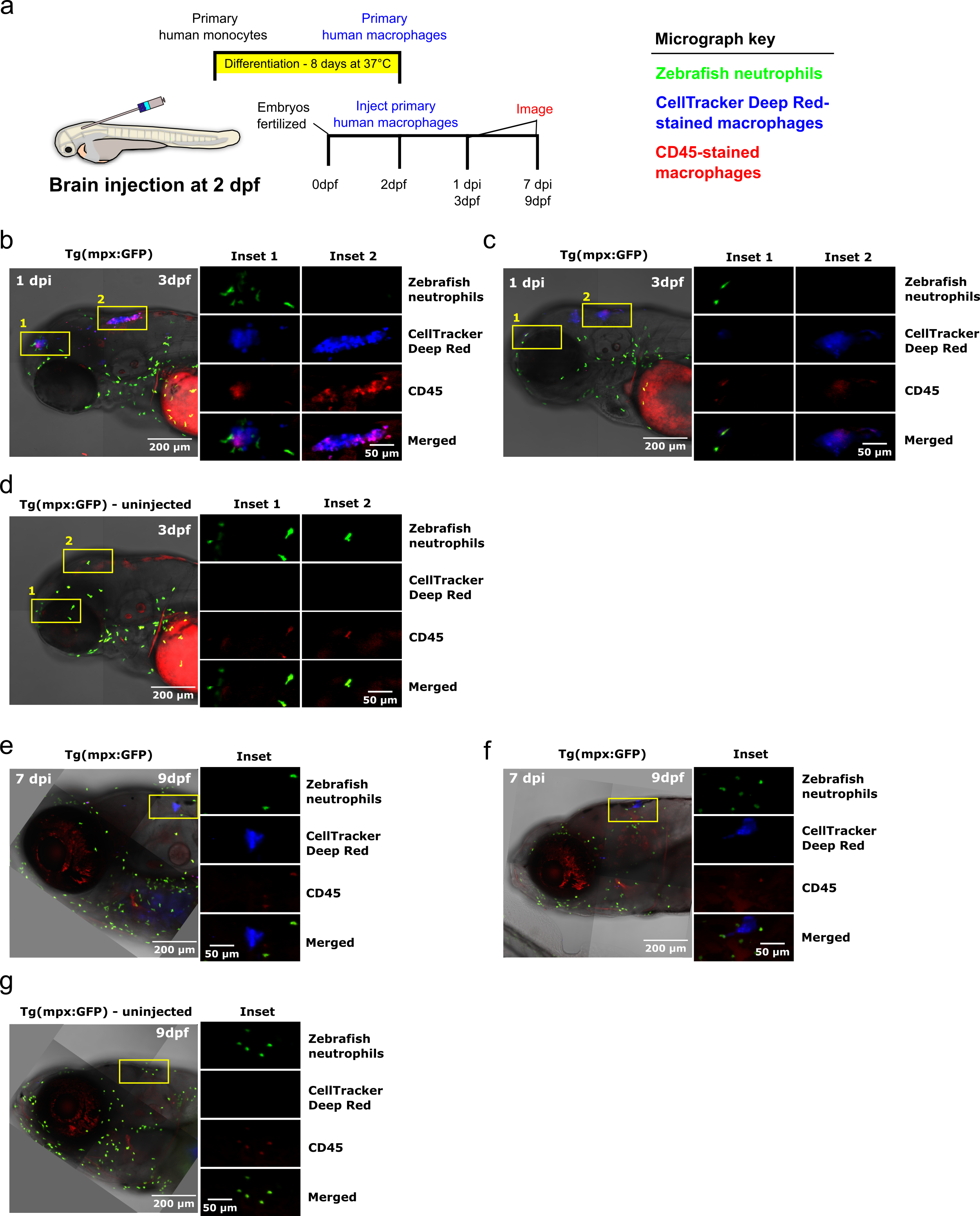


**Supplemental Figure 3- Staining of human macrophages with CD45 antibody.** (a) Schematic of experimental design: primary monocytes were differentiated into macrophages at 37°C before injection into the zebrafish brain at age 2 days post fertilization (dpf) and imaged at 1 and 7 days post injection (dpi). (b,c) Primary human macrophages (blue) were stained with a fluorophore-conjugated antibody against human CD45 (red) and injected into the hind brain of transgenic mpx:GFP (neutrophils-green) zebrafish larvae at 2 dpf. Micrographs of 3D projections showing co-staining of cells with CellTracker Deep Red and the CD45 antibody at 1 dpi. Inset locations are indicated on the overview images. (d) Representative micrograph of 3D projection showing uninjected fish at 3 dpf. Some fluorescence overlap is visible in the CD45 channel (overlap of GFP and AlexaFluor 405). Inset locations are indicated. (e,f) Primary human macrophages (blue) were stained with a fluorophore-conjugated antibody against human CD45 (red) and injected into the hind brain of transgenic mpx:GFP (neutrophils-green) zebrafish larvae at 2 dpf. Micrographs of 3D projections showing co-staining of cells with CellTracker Deep Red and the CD45 antibody at 7 dpi. Inset locations are indicated on the overview images. (g) Representative micrograph of 3D projection showing uninjected fish at 9 dpf. Some fluorescence overlap is visible in the CD45 channel (overlap of GFP and AlexaFluor 405). In 9 dpf images, no fluorescence signal above background was observed in the 405 channel. For all images on a given day, the same acquisition settings and lookup tables were used.


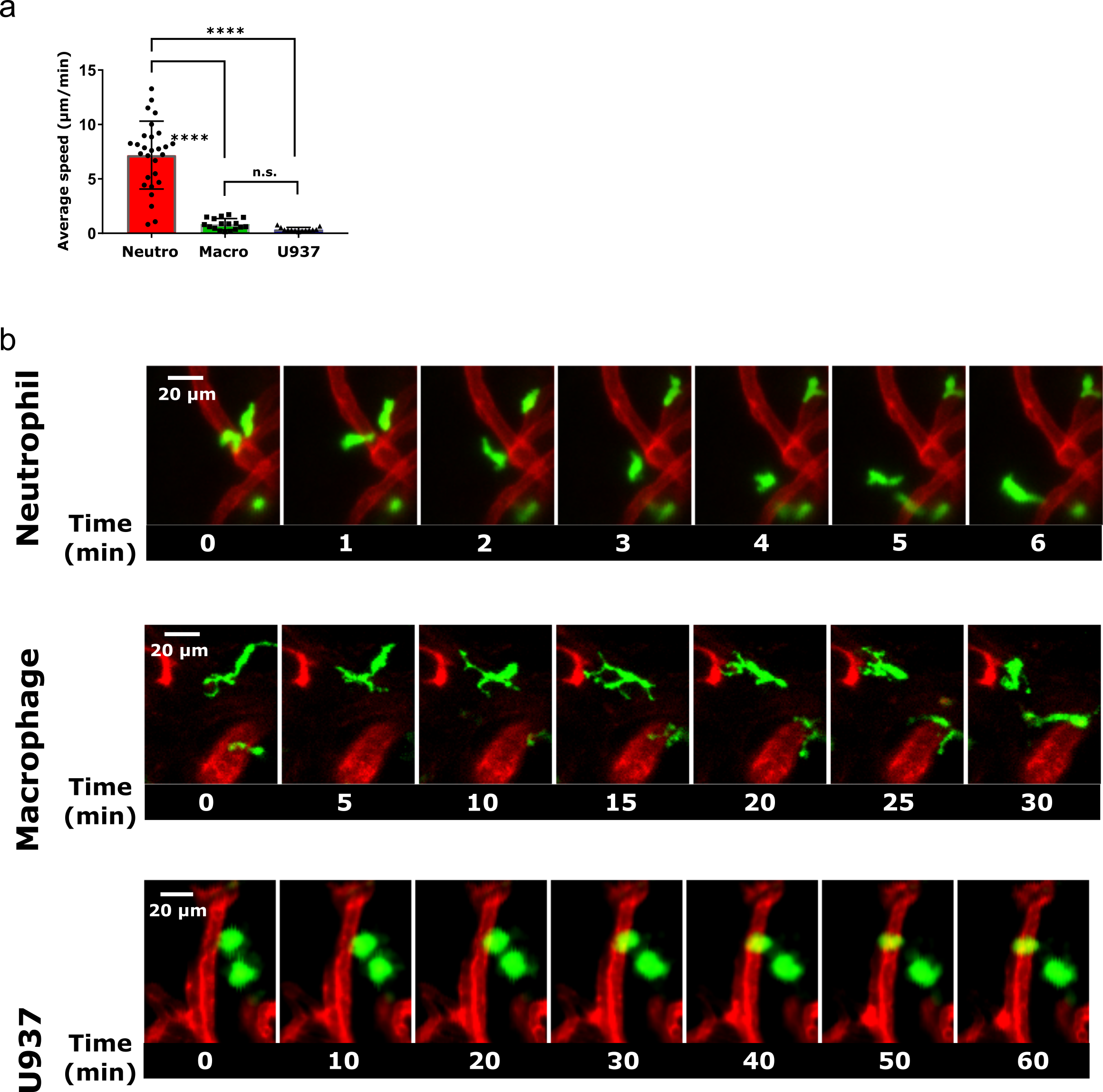


**Supplemental Figure 4- Human immortalized monocytes show similar motilities to zebrafish immune cells *in vivo*.** (a) Plot (mean±SD) of average speeds calculated for brain resident zebrafish macrophages (N=28; fish at 3 dpf), neutrophils (N=17; fish at 3 dpf) and human monocytes (U937; N=13) *in vivo* from one fish per condition. Statistical analysis where **** indicates p<0.0001 by ordinary one-way ANOVA with Tukey’s multiple comparisons post-test. (b) Micrographs show initial position of cells and final position of cells for up to 60 minutes where average speed of human monocytes was calculated from 3D images. Top panel- brain resident zebrafish macrophages, middle panel- brain resident zebrafish neutrophils, and bottom panel- human monocytes injected into the hind brain.

**
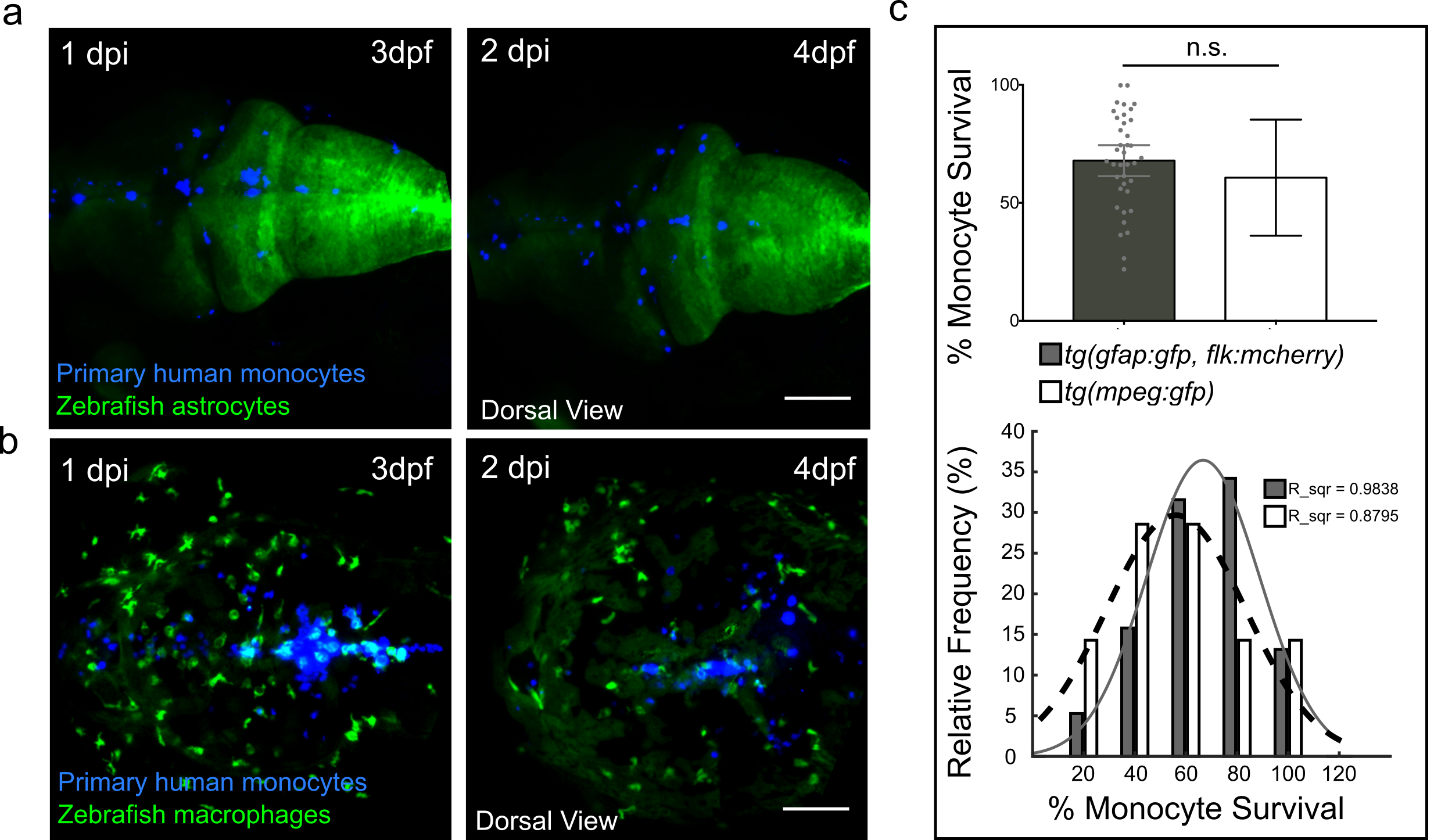
**

**Supplemental Figure 5- Primary human monocytes show similar survival in different strains *in vivo*.** (a) Primary human monocytes (blue) were injected into the hind brain of transgenic zebrafish larave, GFAP:GFP (astrocytes- green) at 2 dpf. Micrographs show representative images where fish was imaged serially over the course of 2 days post injection (2 dpi, 4 dpf) at interval of 24 hrs. (b) Primary human monocytes (blue) were injected into the hind brain of transgenic zebrafish larvae, mpeg:GFP (zebrafish macrophages- green) at 2 dpf. Micrographs show representative images where fish was imaged serially over the course of 2 days post injection (2 dpi, 4 dpf) at interval of 24 hrs. (c) Top panel- Plot (mean±95% CI) of average *in vivo* survival calculated for primary human monocytes obtained from n=38 larvae for GFAP:GFP and n=7 larvae for mpeg:GFP, where the numbers of cells that survived over the course of 2 days were normalized to the initial numbers measured one day post injection. Differences were not significant by unpaired t test with P=0.4083; n.s., not statistically significant. Bottom panel- Graph displays relative distribution of survival based on the initial number of cells.

**
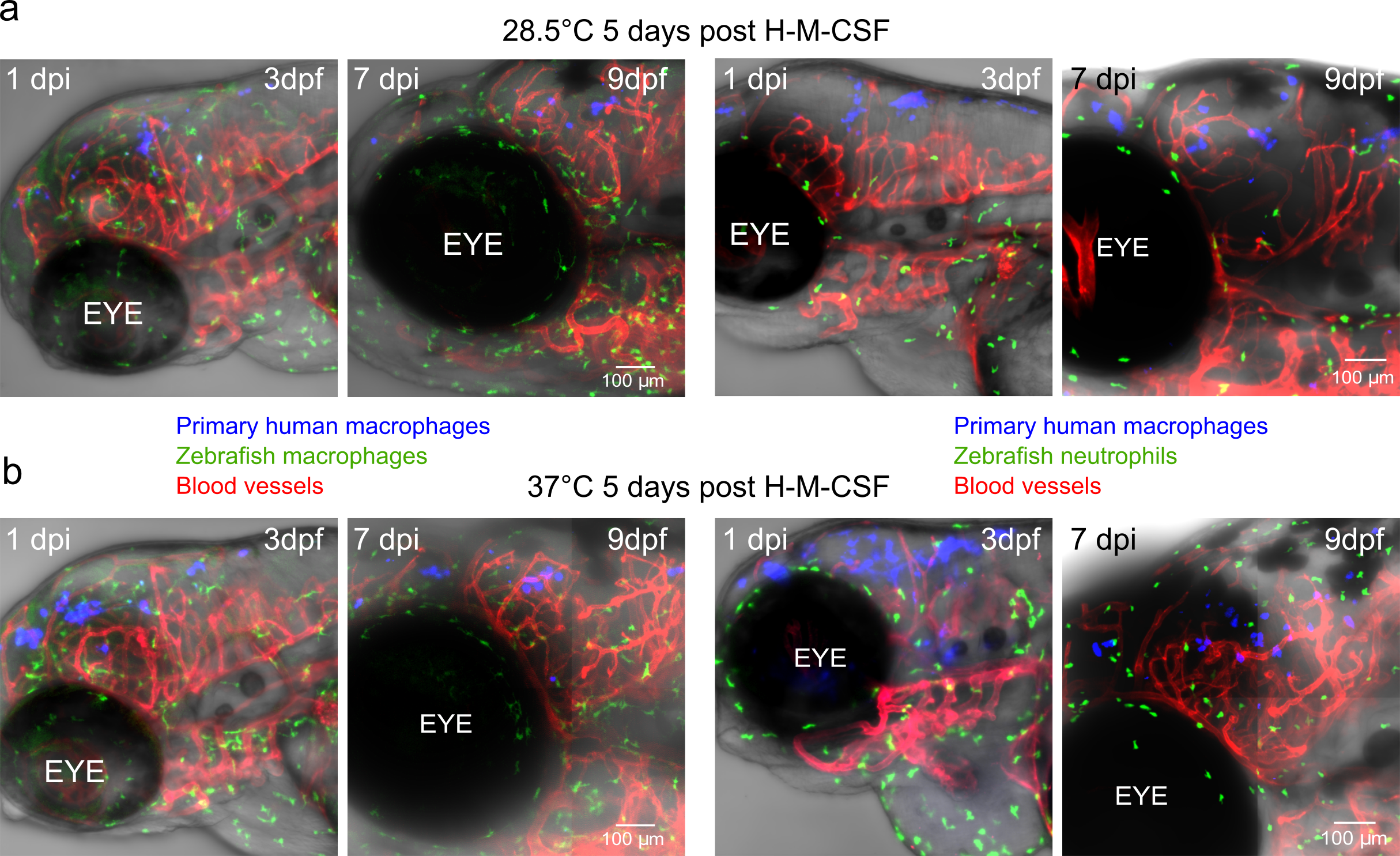
**

**Supplemental Figure 6- Primary human macrophages differentiated at physiological temperatures of the zebrafish or human show similar survival in different strains *in vivo*. Same as Figure 6e,f with bright-field.** Monocytes that had been cultured in the presence of human macrophage colony stimulating factor (H-M-CSF) for five days (b) at physiological temperature of zebrafish (28.5°C) and (c) at physiological temperature of humans (37°C) were differentiated to primary human macrophages. Primary human macrophages (blue) were injected into the hind brain of transgenic zebrafish larvae, mpeg:GFP (macrophages-green)/flk:mCherry (vessels-red) or mpx:GFP (neutrophils-green)/flk:mCherry (vessels-red) at 2 days post fertilization (2 dpf). Micrographs of 3D projections showing distribution and survival of human primary macrophages 1 day post injection (1 dpi) when larvae are 3 dpf and right panel, same fish at 7 dpi (9 dpf).

**Supplemental Movie Legends**

**
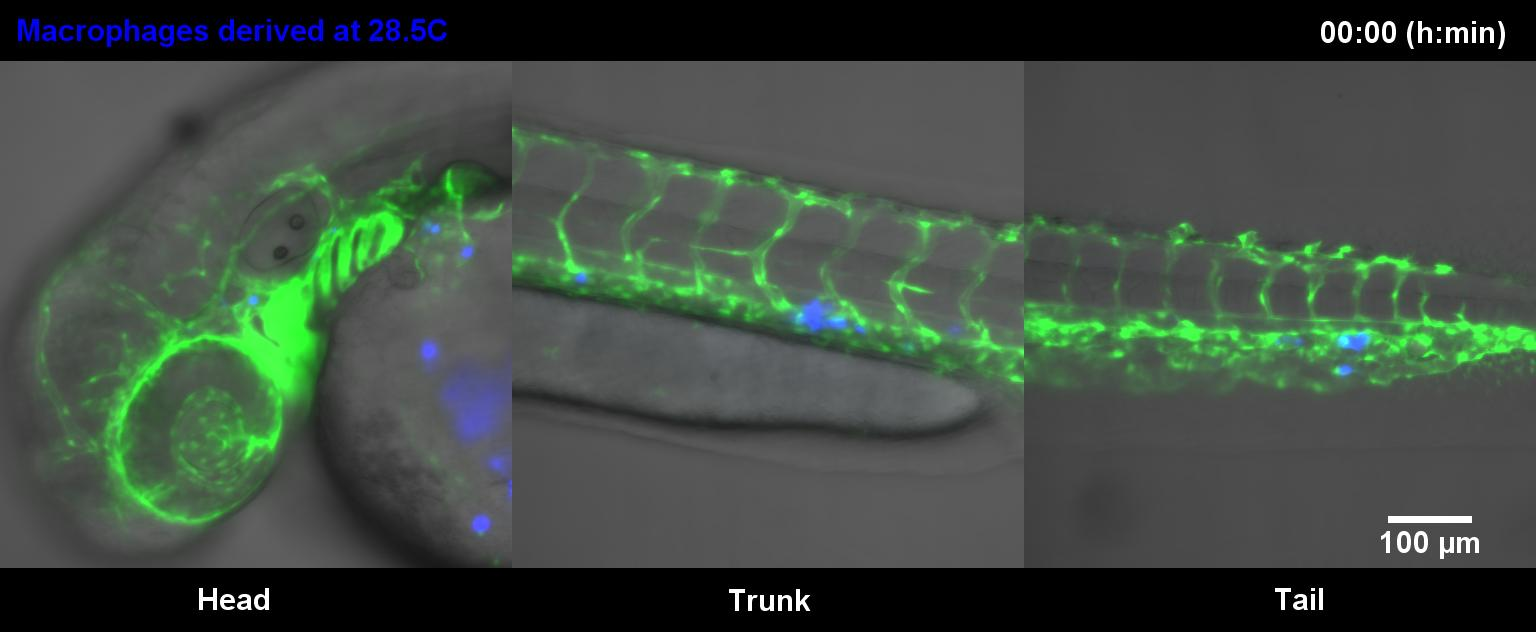
**

**Supplemental Movie 1**- Monocytes that had been cultured in the presence of human macrophage colony stimulating factor (H-M-CSF) for eight days at physiological temperature of zebrafish (28.5°C) were directly injected into the circulation of 2 days post-fertilization (2 dpf) transgenic fli:GFP (vessels-green) zebrafish larvae. Movie shows average intensity projection from 3D time-lapse ~3 hours post-injection for 15 hours at a frame rate of 20 seconds/Zstack (33 planes, 2 µm z step) every 10 minutes. Monocytes are displayed in blue.


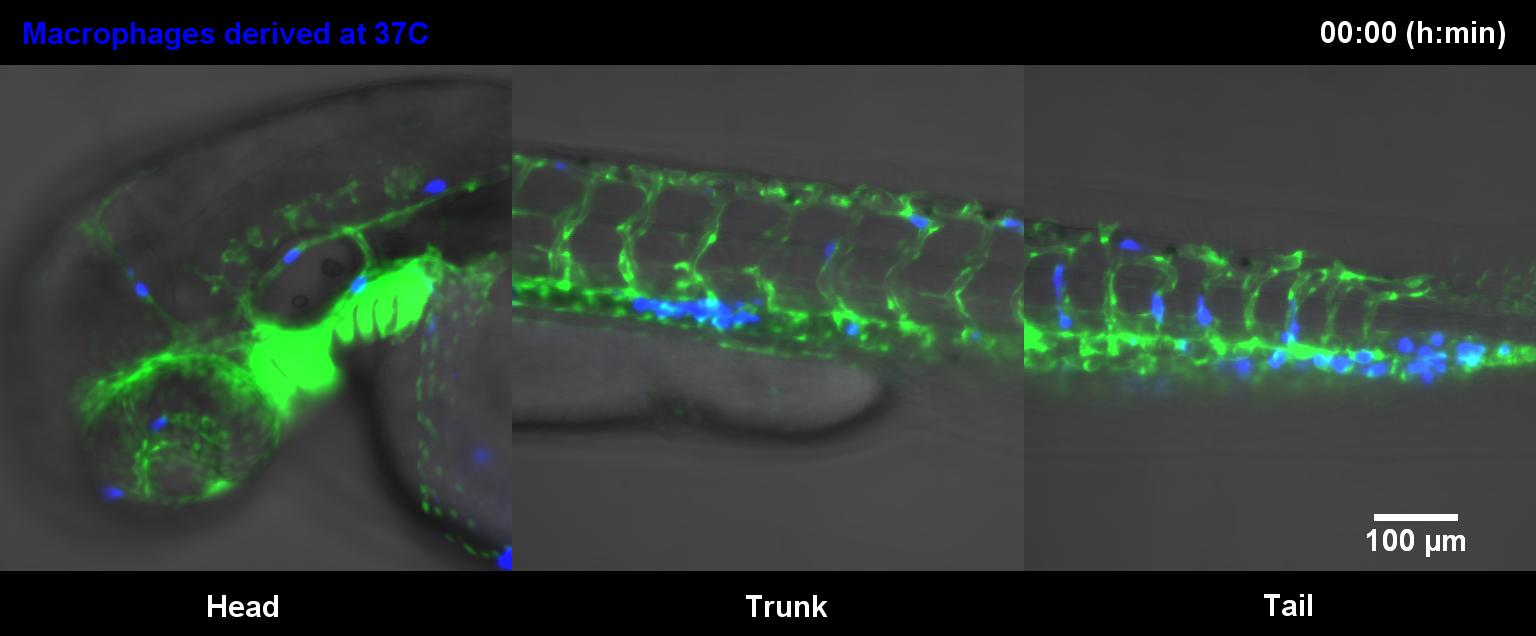


**Supplemental Movie 2**- Monocytes that had been cultured in the presence of human macrophage colony stimulating factor (H-M-CSF) for eight days at physiological temperature of human (37°C) were directly injected into the circulation of 2 days post-fertilization (2 dpf) transgenic fli:GFP (vessels-green) larvae. Movie shows average intensity projection from 3D time-lapse ~3 hours post-injection for 15 hours at a frame rate of 20 seconds/Zstack (33 planes, 2 µm z step) every 10 minutes. Monocytes are displayed in blue.


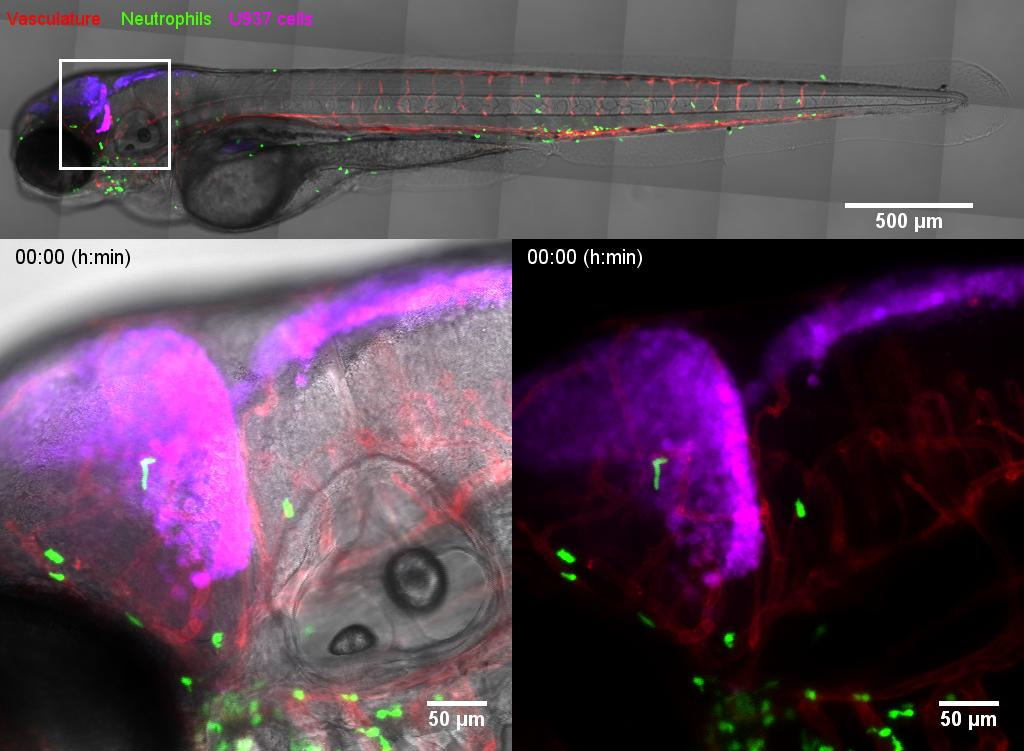


**Supplemental Movie 3**- U937 cells (purple) were directly injected into the hindbrain of 2 days post-fertilization (2 dpf) transgenic mpx:GFP/flk:mcherry (neutrophils-green; vessels-red) larvae. Movie shows average intensity projection from 3D time-lapse ~1 hour post-injection for 3 hours, with images acquired every minute. Location of imaging is indicated on overview image of larva. In bottom panels, left image includes brightfield image. Scales are indicated.


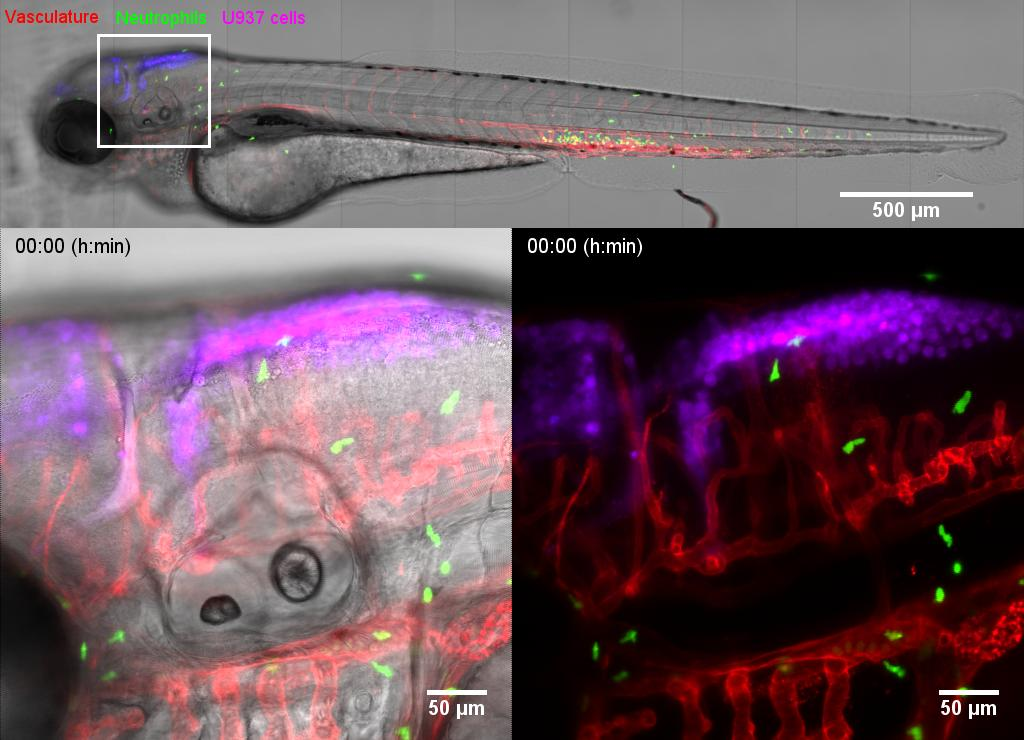


**Supplemental Movie 4**- U937 cells (purple) were directly injected into the hindbrain of 2 days post-fertilization (2 dpf) transgenic mpx:GFP/flk:mcherry (neutrophils-green; vessels-red) larvae. Movie shows average intensity projection from 3D time-lapse ~1 hour post-injection for 3 hours, with images acquired every minute. Location of imaging is indicated on overview image of larva. In bottom panels, left image includes brightfield image. Scales are indicated.


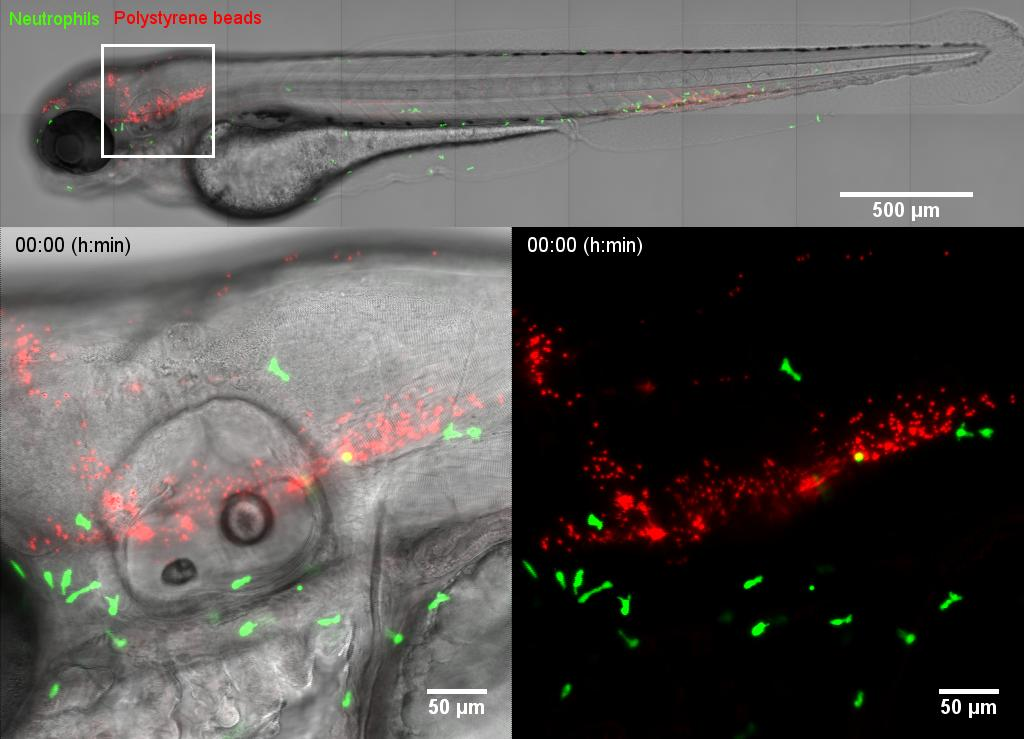


**Supplemental Movie 5**- Polystyrene beads (1 μm-diameter, red) were directly injected into the hindbrain of 2 days post-fertilization (2 dpf) transgenic mpx:GFP (neutrophils-green) larvae. Movie shows average intensity projection from 3D time-lapse ~1 hour post-injection for 3 hours, with images acquired every minute. Location of imaging is indicated on overview image of larva. In bottom panels, left image includes brightfield image. Scales are indicated.


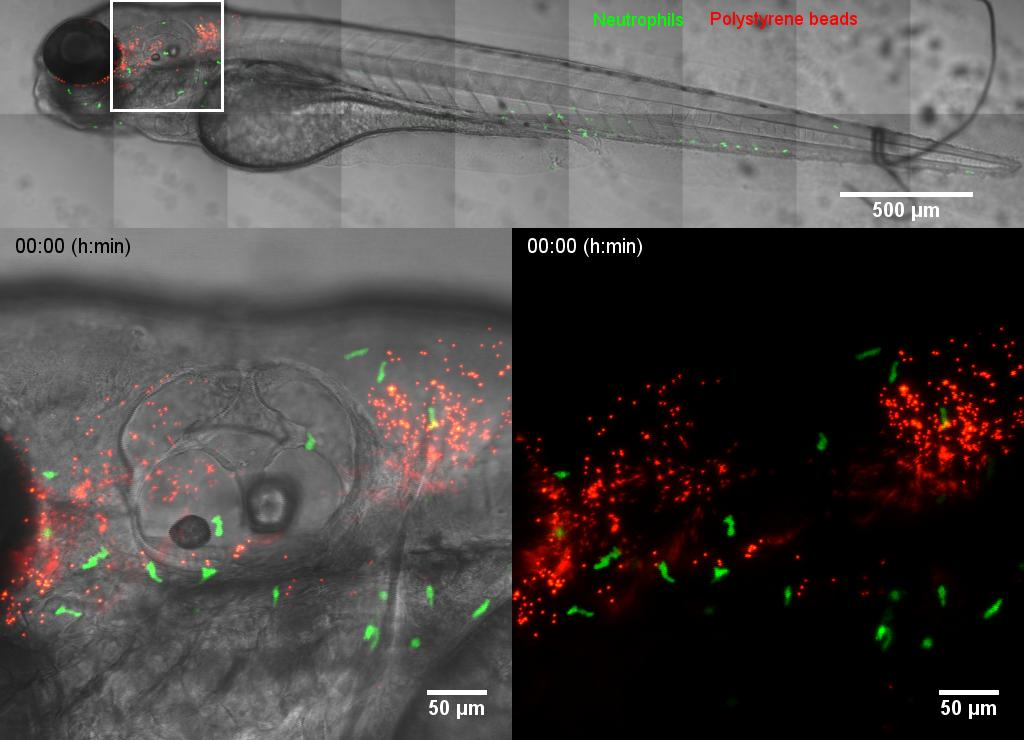


**Supplemental Movie 6**- Polystyrene beads (1 μm-diameter, red) were directly injected into the hindbrain of 2 days post-fertilization (2 dpf) transgenic mpx:GFP (neutrophils-green) larvae. Movie shows average intensity projection from 3D time-lapse ~1 hour post-injection for 3 hours, with images acquired every minute. Location of imaging is indicated on overview image of larva. In bottom panels, left image includes brightfield image. Scales are indicated.
